# Variability, Validity and Operator Reliability of Three Ultrasound Systems for Measuring Tissue Stiffness: A Phantom Study

**DOI:** 10.7759/cureus.31731

**Published:** 2022-11-21

**Authors:** Hafsa Javed, Samson O Oyibo, Abdulrahman M Alfuraih

**Affiliations:** 1 General Medicine, Peterborough City Hospital, Peterborough, GBR; 2 Endocrinology, Peterborough City Hospital, Peterborough, GBR; 3 Radiology, Prince Sattam Bin Abdulaziz University, Al-Kharj, SAU

**Keywords:** phantom, tissue stiffness, shear wave elastography, reliability, validity

## Abstract

Introduction

Ultrasound elastography is a method of measuring soft tissue stiffness to detect the presence of pathology. There are several ultrasound elastography devices on the market. The aim of this study was twofold. Firstly, to determine the validity of three different ultrasound systems used to measure tissue stiffness. Secondly, to determine the operator reliability and repeatability when using these three systems.

Materials and methods

Two observers undertook multiple stiffness measurements from a phantom model using three different ultrasound systems; the LOGIQ E9, the Aixplorer, and the Acuson S2000. The phantom model had four cylindrical-shaped inclusions (Type 1-4) of increasing stiffness values and diameter embedded within. The background phantom stiffness was fixed. The mean, standard deviation, and coefficient of variation (CV) were calculated from measured stiffness readings per diameter per inclusion. Intra-observer variability was assessed. The validity of the measured stiffness value was assessed by calculating the difference between the measured elasticities and actual phantom elasticities. Bland-Altman plots with limits of agreement were used to display the inter-observer agreement. The intraclass correlation coefficients (ICC) were used to measure intra-observer, inter-observer, and inter-system reliability.

Results

Each observer undertook 1020 measurements. All three systems generally underestimated the stiffness values for the inclusions; the higher the actual stiffness value, the more significant the underestimation. The percentage difference between measured stiffness and actual stiffness varied from -79.1% to 12.7%. The intra-observer variability was generally less than 5% for observers using the LOGIQ E9 and the Aixplorer systems but more than 10% over the stiffer inclusions (Types 3 and 4) for the Acuson system. There was 'almost perfect' intra-observer reliability and repeatability for both the LOGIQ E9 and the Aixplorer systems; this was 'moderate' for the Acuson system over specific inclusions. For all systems, there was 'almost perfect' inter-observer reliability and repeatability between Observer A and Observer B. The inter-system reliability and repeatability were 'almost perfect' between the LOGIQ E9 system and the Aixplorer system but 'poor' and 'moderate' when the Acuson system was matched with the LOGIQ E9 system and the Aixplorer system, respectively.

Conclusion

This study has demonstrated that the Acuson, LOGIQ E9, and Aixplorer ultrasound systems have low variability, high reproducibility, and good intra-observer and inter-observer reliability when used to measure tissue stiffness. However, they all underestimated the stiffness values during this in vitro study. This study also revealed that not all ultrasound systems are comparable when measuring tissue stiffness, with some having better inter-system reliability than others. Ongoing standardization of technology is required at the manufacturer level.

## Introduction

Ultrasound elastography or shear wave elastography (SWE) is an ultrasound-applied imaging technique used to determine tissue stiffness due to underlying disease. This non-invasive technology allows healthcare professionals to observe and quantify signs of abnormal tissue stiffness in deeper structures [1]. Tissue fibrosis and tumors can lead to variations in tissue stiffness compared to the normal elasticity of the surrounding tissue [2]. Ultrasound elastography is inexpensive, easily accessible, portable, and clinically versatile, and its use continues to aid in early diagnosis and prompt treatment of fibrosis and tumors [2].

SWE technology employs the use of stimulated shear waves to determine Young's modulus (stress to strain ratio) of tissues in kilopascals (kPa): the higher the value, the higher the stiffness [2-4]. An ultrasound device sends impulses to the underlying tissue of interest, generating shear waves. Shear waves travel perpendicular to the original wave and are picked up by the ultrasound system software to produce an elasticity map of tissue stiffness [2-4]. Three types of shear wave imaging technology used in clinical applications are point SWE, one-dimensional transient SWE, and two-dimensional SWE [2-4]. SWE differs from strain (compressive) elastography, where stress is applied by repeated manual compression of the ultrasound transducer. The amount of lesion deformation relative to the surrounding normal tissue is measured and displayed in color [5]. 

There are several ultrasound systems on the market. Much of the technology and mechanisms behind SWE are patented and, therefore, inaccessible to researchers and clinicians [3]. However, only a few studies have explored the inter-system accuracy and reliability of different ultrasound systems [3,6]. Additionally, while previous studies have investigated intra- and inter-observer reliability, agreement, and validity [7-9], only some have explored the disparities in reliability and variability of measurements about the size of the specific region of interest (ROI). This data is essential for healthcare providers when deciding which ultrasound system to purchase for measuring tissue stiffness.

Therefore, the aim of this study was twofold. Firstly, to determine the validity of three different ultrasound systems by comparing measured and actual stiffness values. Secondly, to determine operator reliability by calculating the ultrasound systems' intra-observer, inter-observer, and inter-system reliability.

## Materials and methods

The ultrasound systems

The three ultrasound systems used for this study were the Acuson S2000 (Siemens, Germany), the Aixplorer (SuperSonic Imagine, France), and the LOGIQ E9 (General Electric, UK). The Aixplorer and LOGIQ E9 systems use two-dimensional SWE technology to measure kilopascals' stiffness (kPa). The Acuson S2000 system uses point SWE technology and measures the shear wave velocity in meters per second (m/s): a conversion factor was used to change this to kPa. The manufacturer-recommended brightness mode (B-mode) and elastography settings were used to ensure readings' consistency and methodology standardization. All three systems had similar linear probes with similar frequency bandwidths to allow comparison between the systems.

The phantom

**Figure 1:**
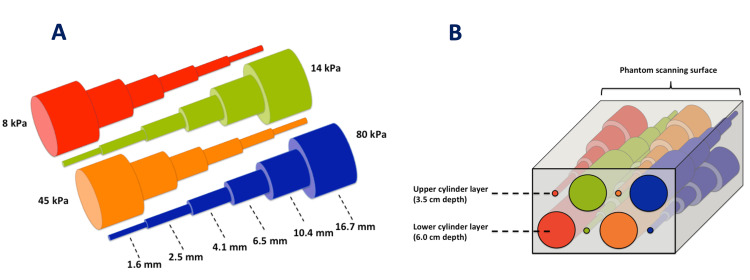
Schematic diagram of the elasticity QA phantom and inclusions A: The four inclusions with reducing diameter; Type 1: Red (8kPa); Type 2: Green (14 kPa); Type 3: Orange (45 kPa); Type 4: Blue (80kPa), B: The phantom layout showing the four inclusions at 3.5 cm and 6.0 cm depth from the scanning surface. Reproduced with permission from Carlsen JF et al. A comparative study of strain and shear-wave elastography in an elasticity phantom. AJR Am J Roentgenol 2015;204:W236-242, Copyright© 2013-2020, American Roentgen Ray Society.

A phantom model was used to represent human tissue to obtain stiffness values. The Elasticity QA phantom (Model 049, Zerdine®) was loaned from Computerized Imaging Reference Systems Company (CIRS, Virginia, USA) for this study (Figure 1). This phantom contains four cylindrical inclusions of various stiffness values (Type 1: 8 kPa; Type 2: 14 kPa, Type 3: 45 kPa, and Type 4: 80 kPa) at a depth 3.5 cm and four identical cylindrical inclusions at a depth of 6.0 cm. Each cylindrical inclusion is further divided into six partitions of decreasing diameters (16.7, 10.4, 6.5, 4.1, 2.5, and 1.6 mm). The background stiffness value of the phantom was 25 kPa. The five largest partitions of the cylindrical inclusion were included in this study as the smallest diameter proved challenging to quantify and visualize in B-mode imaging [4]. The ultrasound probes could not penetrate the inclusions at 6.0 cm depth, so only the inclusions at 3.5 cm depth were used in this study.

Study design

Observers A and B, blinded to each other's readings, undertook a set of measurements on the elastography phantoms using a single linear probe. Measurements were done on five partitioned sites on each of the four inclusions, totaling 20 sites. Each observer was instructed to do 20 repeat measurements per site, equating to 400 readings per ultrasound system used. Therefore, 1200 readings per observer were expected after using the three systems. In order to standardize the method, the ROI for each diameter was predetermined and remained the same for the Aixplorer and LOGIQ E9 ultrasound systems. However, a rectangular fixed ROI was used for the Acuson S2000 system. Each reading was taken after two frame rates after the probe was positioned, and the elastography function was selected and recorded.

Statistical analysis

Statistical analyses were performed using IBM SPSS Statistics for Windows version 22.0 (IBM Corp., Armonk, NY, USA). Each set of 20 measured stiffness readings attained per diameter per cylindrical inclusion was averaged to calculate the mean, standard deviation (SD), and coefficient of variation (CV). Intra-observer variability was assessed by the coefficient of variation (CV). The CV was determined by dividing the standard deviation by the mean value: the lower the CV, the lower the variability. A CV less than 0.1 (10%) was considered low variability. The validity of the measured stiffness value was assessed by calculating the mean difference (and percentage mean difference) between the measured stiffness values and actual phantom stiffness values. Bland-Altman plots with limits of agreement (95%, 1.96 SD) were used to display inter-observer agreement and reliability and the difference between observers against the mean to display systematic variation [10]. Intraclass correlation coefficients (ICC) were used to measure intra-observer, inter-observer, and inter-system reliability/agreement. An ICC reading of 0.00-0.20 indicated 'poor' reliability, 0.21-0.41 indicated 'fair' reliability, 0.41-0.60 indicated 'moderate' reliability, 0.61-0.80 was interpreted as 'substantial' reliability, and a reading greater than 0.80 indicated 'almost perfect' reliability or agreement [11].

## Results

A total of 2080 stiffness measurements were taken for this study, 1040 per observer. Each observer took 400 measurements of the phantom using the LOGIQ E9 system, 400 using the Aixplorer system, and 240 using the Acuson S2000 system. The Acuson system could not obtain stiffness measurements from the two smallest diameters (4.1 mm and 2.5 mm), so these were omitted. The Acuson system could not obtain measurements accurately at the 3.0 cm depth, the standardized parameter across the three systems. Therefore, a depth of 2.7 cm (reaching the three largest diameters) was used for the Acuson system.

Table 1 reports the mean measured stiffness readings, a difference from actual values, and coefficient of variation (CV) values for each inclusion type for each of the three ultrasound systems per observer. A more extensive table (Table 5) displaying the values for each ROI for each inclusion can be found in the appendix. Both observers underestimated the elasticity values across all inclusions and all systems, except for the two smallest diameters on inclusion Type 1 (8 kPa), which were only measured using the Aixplorer and LOGIQ E9 systems. Observer A reported higher stiffness readings than Observer B throughout the study.

**Table 1:** Mean values and measures of variation for stiffness measurements for all three ultrasound systems Mean (SD) stiffness values in kPa, actual difference and the percentage difference between measured values and actual values, and the coefficient of variation (CV) for both Observers A and Observer B across all three systems and all four types of inclusions in the phantom. Mean difference = (actual stiffness - mean measured stiffness), percentage mean difference (%) = [(mean measured stiffness ÷ actual stiffness) x 100] - 100.

Phantom Inclusion (stiffness)	Categories		Ultrasound system		
			LOGIQ E9	Aixplorer	Acuson S2000
Type 1 (8 kPa)	Observer A	Mean (SD) kPa	8.25 (0.32)	9.01 (0.15)	5.43 (0.22)
		CV	0.04	0.02	0.04
		Mean Diff (%)	-0.25 (3.1%)	-1.01 (12.7%)	2.57(-32.2%)
	Observer B	Mean (SD) kPa	8.19 (0.35)	8.40 (0.12)	4.98 (0.1)
		CV	0.05	0.01	0.02
		Mean Diff (%)	-0.19 (2.4%)	-0.40 (5.0%)	3.02 (-37.7%)
Type 2 (14 kPa)	Observer A	Mean (SD) kPa	11.20 (0.21)	11.54 (0.27)	10.19 (0.19)
		CV	0.02	0.02	0.02
		Mean Diff (%)	2.80 (-20.0%)	2.46 (-17.6%)	3.81 (-27.2%)
	Observer B	Mean (SD) kPa	10.6 (0.3)	10.7 (0.1)	9.33 (0.2)
		CV	0.03	0.01	0.02
		Mean Diff (%)	3.4 (-24.3%)	3.3 (-23.6%)	4.67 (-33.3%)
Type 3 (45 kPa)	Observer A	Mean (SD) kPa	23.08 (0.38)	23.78 (0.22)	17.58 (1.0)
		CV	0.02	0.01	0.06
		Mean Diff (%)	21.92 (-48.7%)	21.22 (-47.1%)	27.42 (-60.9%)
	Observer B	Mean (SD) kPa	21.88 (0.34)	23.39 (0.38)	17.42 (1.26)
		CV	0.02	0.02	0.07
		Mean Diff (%)	23.13 (-51.4%)	21.61 (-48.0%)	27.58 (-61.3%)
Type 4 (80 kPa)	Observer A	Mean (SD) kPa	35.99 (1.2)	42.01 (0.66)	14.76 (1.2)
		CV	0.03	0.02	0.14
		Mean Diff (%)	44.01 (-55.0%)	37.99 (-47.5%)	65.24 (-81.5%)
	Observer B	Mean (SD) kPa	35.80 (0.7)	40.46 (0.62)	16.71 (7.0)
		CV	0.02	0.02	0.42
		Mean Diff (%)	44.20 (-55.2%)	39.54 (-49.4%)	63.29 (-79.1%)

Intra-observer variability, validity, and accuracy

The intra-observer variability was generally low for both observers, indicated by a CV range of 0.01-0.05. The lowest variability was noted while using the Aixplorer ultrasound system, and the highest (CV more than 0.1) was noted while using the Acuson system, especially when measuring over the Type 3 and Type 4 inclusions.

Both observers reported an extensive range in the mean differences across all systems, indicating significant measurement biases and low validity and accuracy. The percentage difference between measured stiffness and actual stiffness varied from -79.1% to 12.7%. When using the LOGIQ E9 and Aixplorer systems to measure the Type 1 inclusion, observers overestimated the stiffness values. Stiffness values were underestimated using all three systems over all other ROI; the higher the actual stiffness value of the inclusion, the greater the underestimation. The Acuson system underestimated the stiffness value at every ROI and had the largest percentage differences between measured and actual stiffness values as the inclusion stiffness increased from Type 1 to Type 4.

Inter-observer agreement

**Figure 2:**
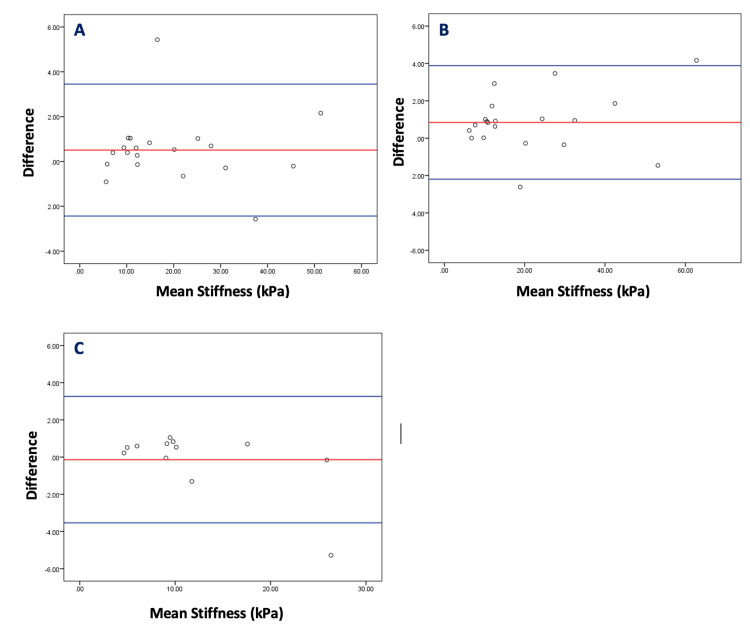
Bland Altman plots for inter-observer agreement and limits of agreement For each graph, the Y-axis is the difference between paired stiffness measurements from Observer A and Observer B, while the X-axis is the mean of the paired stiffness measurements from Observer A and Observer B. The red central line shows the mean difference in stiffness values between both observers. The blue lines indicate the upper and lower 95% limits of agreement for (A) LOGIQ E9, (B) Aixplorer, and (C) Acuson.

Inter-observer agreement was evaluated using Bland Altman plots and the 95% limits of agreement. Figure 2 shows the plots for the 20 paired stiffness readings (calculated means), each for the LOGIQ E9 and the Aixplorer systems and 12 paired readings for the Acuson system. The plots show the variation and agreement of the mean of the measurements between Observer A and Observer B. The greater the number of plots that fell within the 95% limits of agreement, the greater the inter-observer agreement. For the LOGIQ E9 system, the mean difference in stiffness values between both observers was 0.50 (-2.43 - 3.45) kPa, and 18 out of 20 (90%) plots fell within the 95% limits of agreement, indicating good agreement between both observers. For the Aixplorer system, the mean difference in stiffness values between both observers was 0.84 (-2.20 - 3.88) kPa, and 18 out of 20 (90%) plots fell within the 95% limits of agreement, also indicating good agreement between both observers. For the Acuson system, the mean difference in stiffness values between both observers was 0.14 (-3.54 - 3.26) kPa, and 11 out of 12 (91.7%) plots fell within the 95% limits of agreement, indicating good agreement between both observers. The mean difference for the Acuson system was the closest to zero yet had the greatest variation between the limits of agreement.

Intra-observer reliability

ICC was calculated to determine intra-observer reliability. Table 2 shows the ICC values obtained for each inclusion and each ultrasound system per observer. Both observers demonstrated 'almost perfect' agreement between repeated measurements when using the LOGIQ E9 and Aixplorer systems (ICC range: 0.955-0.999). While using the Acuson system, Observer A demonstrated 'almost perfect' agreement, apart from measuring over the Type 2 inclusion, when the agreement fell to 'moderate' (ICC: 0.528). Furthermore, while using the Acuson system, Observer B demonstrated an 'almost perfect' agreement, apart from measuring over the Type 4 inclusion, when the agreement fell to 'moderate' (ICC: 0.476).

**Table 2:** Intra-observer reliability The ICC values are reported for both Observer A and Observer B for measurements over each inclusion for each ultrasound system. The lower and upper 95% confidence intervals are also reported.

Ultrasound system	Phantom Inclusion	ICC	95% Confidence Intervals		ICC	95% Confidence Intervals	
		Observer A	Lower	Upper	Observer B	Lower	Upper
LOGIQ E9	Type 1	0.989	0.967	0.999	0.973	0.923	0.997
	Type 2	0.955	0.877	0.994	0.924	0.804	0.990
	Type 3	0.995	0.987	0.999	0.998	0.995	1.000
	Type 4	0.992	0.977	0.999	0.997	0.992	1.000
Aixplorer	Type 1	0.997	0.990	1.000	0.997	0.990	1.000
	Type 2	0.971	0.919	0.996	0.985	0.956	0.998
	Type 3	0.999	0.998	1.000	0.998	0.994	1.000
	Type 4	0.999	0.996	1.000	0.998	0.995	1.000
Acuson S2000	Type 1	0.923	0.743	0.998	0.977	0.911	0.999
	Type 2	0.528	0.192	0.979	0.861	0.598	0.996
	Type 3	0.984	0.937	1.000	0.970	0.887	0.999
	Type 4	0.913	0.718	0.998	0.476	0.157	0.974

Inter-observer reliability

Table 3 shows the ICC value and, thus, inter-observer reliability when using each system. The ICC values ranged from 0.975 to 0.993, indicating an 'almost perfect' agreement between observers A and B when using all three systems.

**Table 3:** Inter-observer reliability The ICC values are reported for both Observer A and Observer B for each ultrasound system. The lower and upper 95% confidence intervals are also reported.

Ultrasound system	Inter-observer reliability (ICC)	95% Confidence Intervals	
		Lower	Upper
LOGIQ E9	0.993	0.983	0.997
Aixplorer	0.994	0.982	0.998
Acuson S2000	0.975	0.916	0.993

Inter-system reliability

The ICC was used to determine inter-system reliability and repeatability. Table 4 shows the reliability or agreement between the different ultrasound systems for each observer. Each system was compared with one another at a time for each observer. For both Observers A and B, there was 'almost perfect agreement between the LOGIQ E9 and the Aixplorer systems. However, the Acuson system demonstrated 'poor' agreement and 'fair' agreement with the LOGIQ E9 system with ICC values of 0.202 and 0.238, in the hands of Observer A and Observer B, respectively. Additionally, the Acuson system demonstrated 'fair' agreement and 'moderate' agreement with the Aixplorer system with ICC values of 0.341 and 0.463, in the hands of Observer A and Observer B, respectively.

**Table 4:** Inter-system reliability The ICC values are reported for each ultrasound system compared against one another for both Observers A and B. The lower and upper 95% confidence intervals are also reported.

Observer	LOGIQ E9 vs Aixplorer			LOGIQ E9 vs Acuson S2000			Aixplorer vs Acuson S2000		
	ICC	95% Confidence Intervals		ICC	95% Confidence Intervals		ICC	95% Confidence Intervals	
		Lower	Upper		Lower	Upper		Lower	Upper
A	0.963	0.890	0.986	0.202	-0.440	0.687	0.341	-0.299	0.757
B	0.977	0.919	0.990	0.238	-0.362	0.699	0.463	-0.117	0.808

## Discussion

Ultrasound elastography has been widely introduced into the clinical environment. Studies and clinical use have extended to carotid lesions, breast lesions, pancreatic cysts, myocardial stiffness, liver lesions, testicular torsion, and malignant thyroid nodules, to name a few [12-18]. Thus, it is important to investigate and compare the performance of elastography systems that are commercially available for clinical use.

This study aimed to investigate the validity and operator validity across three different ultrasound systems. Two independent observers (Observer A and Observer B) measured stiffness/stiffness levels of four different inclusions in a single phantom model using the LOGIQ E9 system, the Aixplorer system, and the Acuson system. Within the phantom were four inclusions (with increasing stiffness, Type 1-4); each inclusion had five partitions with increasing diameter. The LOGIQ E9 and the Aixplorer systems slightly overestimated the stiffness values for the softest inclusion (Type 1) at its smallest diameter; the Acuson system failed to get measurements over the two smallest diameter sections of all inclusions. All three systems underestimated the stiffness values for the inclusions at all other ROIs when used by both observers; the higher the actual stiffness value of the inclusion, the greater the underestimation. The Acuson system underestimated the stiffness value by the greatest degree, by up to 80% for the stiffest (Type 4) inclusion. Observer A consistently had slightly higher stiffness values than Observer B for each measurement. The Acuson system could not get a reading for the two smallest diameters on the softest inclusion (Type 1). The intra-observer variability was generally low for both observers, especially when using the LOGIQ E9 system and the Aixplorer system, with CV less than 5%. At the same time, the variability was higher with the Acuson system (CV more than 10% when measuring over the stiffer Type 3 and Type 4 inclusions. There was a good inter-observer agreement when using all three systems. There was 'almost perfect' intra-observer reliability and repeatability for all measurements using the LOGIQ E9 and the Aixplorer systems. However, this dropped to 'moderate' reliability for the Acuson system when used over the Type 2 inclusion by Observer A and the Type 4 inclusion by Observer B. For all systems, there was 'almost perfect' inter-observer reliability and repeatability between Observer A and Observer B. The inter-system reliability and repeatability were 'almost perfect' between the LOGIQ E9 system and the Aixplorer system. However, the inter-system reliability was 'poor' and 'moderate' when the Acuson system was matched with the LOGIQ E9 and the Aixplorer systems, respectively.

The stiffness of the phantom was fixed at 25 kPa. It is possible that the ultrasound systems were unable to differentiate the background stiffness from the stiffness of the smallest diameter regions of the softest inclusion (Type 1), thus overestimating the stiffness value of the softest inclusion. A similar explanation would account for the marked underestimation of stiffness values for all the stiffer inclusions, especially those with actual stiffness values greater than the background (phantom stiffness). Previous research has questioned the validity of the phantoms being used for such studies [7].

The stiffness readings from the Acuson system were the least accurate of the three systems, with the greatest underestimation and highest variability. These inaccuracies have been highlighted in other studies using this system [4,6,9,19]. The Acuson system measured share wave velocity in meters per second (instead of stiffness), and results were converted to kPa before analysis. The conversion formula uses the density of the medium. The manufacturer did not provide the phantom specifications for density, so density was assumed to be 1g/cm3, the density of soft tissue, as this is a tissue-mimicking phantom. As this was an assumption, this may have led to some inaccuracies in the readings provided by the Acuson system. Additionally, the point SWE technology employed by the Acuson system may not be optimized for harder inclusions, thus producing error readings [4,6].

This study demonstrated high intra-observer and inter-observer reliability/agreement. Previous phantom studies have also reported comparable high intra-observer and inter-observer agreement [6,7,20]. There was also a good inter-system agreement between the LOGIQ E9 and the Aixplorer system but reduced agreement with the Acuson system. A previous study found great disparities between measurements obtained using the Aixplorer (Supersonic Imagine) versus the Siemens Acuson S2000, as found in our study. However, that study used a phantom with only one stiffness value [21].

This result has profound clinical importance, as they highlight that not all ultrasound systems are equivalent when measuring tissue stiffness. Ultrasound elastography is widely employed for diagnostic purposes; therefore, treatment, response, and prognosis decisions are based on such platforms. The results may differ if patients move between healthcare institutions employing alternative ultrasound systems; therefore, clinical decision-makers must consider this during clinical practice. Conversion formulae have been suggested to adapt the measurements between ultrasound systems to aid comparability [3].

Study limitations

The probes used in this study could not read at 6 cm depth as originally intended for this study, and therefore, depth analysis was excluded. All probes used were linear in design, but their frequency range was not identical; their frequencies varied from 2-10 MHz, which may have introduced some bias into the stiffness readings. However, the frequencies for the Aixplorer, LOGIQ E9, and the Acuson systems are comparable at 10 MHz, 9 MHz, and 9 MHz, respectively, so the effect of frequency may be less profound. To standardize the method, the ROI for each diameter was predetermined, and the readings were recorded after two frame rates. However, in practice, this was only partially possible. The ROI was easily specified to Aixplorer before readings, but for the LOGIQ E9, the ROI had to be manually circumscribed for every reading. The ROI was rectangular by default on the SA system. Therefore, these manipulations may have added errors or bias to the readings. Tissue-mimicking phantoms do not possess the viscoelastic properties of actual tissue. Therefore the results obtained do not necessarily fully reflect the stiffness readings in vivo in the clinical environment [7].

Further research

The phantom's stiffness may affect the inclusions' stiffness readings. The validity of the phantoms used in studies could be evaluated by employing multiple phantoms and observers and comparing more stiffness readings. Few studies have compared the reliability and accuracy of results obtained at different depths by ultrasound systems in a phantom. Therefore, depth analysis in vivo and in vitro is recommended for future research. More in vivo studies are required to determine actual tissue stiffness at different sites so accurate phantom can be developed for repeatability and variability studies of stiffness reading.

## Conclusions

This study has demonstrated that the Acuson, LOGIQ E9, and Aixplorer ultrasound systems have low variability, high reproducibility, and good intra-observer and inter-observer reliability when measuring tissue stiffness. However, they all underestimate the true stiffness values during in vitro studies, requiring a correction formula. This study also revealed that not all ultrasound systems are comparable when used to measure tissue stiffness, with some having better inter-system reliability than others. Therefore, applying the readings from one machine to another in the clinical environment may not be accurate. So ultrasound systems require standardization between manufacturers to propel them into absolute clinical integration. We hope this study not only contributes to the literature but also encourages further research into this exciting topic.

## Appendices

**Table 5:** Mean values and measures of variation for elasticity measurements for all three ultrasound systems, phantom inclusions, and observers Mean (SD) stiffness values in kPa, actual difference and the percentage difference between measured values and actual values, and the coefficients of variation (CV) for both Observers A and Observer B across all three systems and all four types of inclusions in the phantom. Mean difference = (actual stiffness - mean measured stiffness), percentage mean difference (%) = [(mean measured stiffness ÷ actual stiffness) x 100] - 100. Mean difference = (actual stiffness - mean measured stiffness), Percentage mean difference (%) = [(mean measured stiffness ÷ actual stiffness) x 100] - 100.

Phantom inclusion (stiffness)	Diameter	Categories		LOGIQ E9	Aixplorer	Acuson S2000
Type 1 (8 kPa)	2.5	Observer A	Mean (SD) kPa	12.23 (0.30)	13.15 (0.24)	Nil result
			CV	0.03	0.02	Nil result
			Mean Diff (%)	-4.23 (52.9%)	-5.15 (64.4%)	Nil result
		Observer B	Mean (SD) kPa	12.36 (0.42)	12.24 (0.25)	Nil result
			CV	0.03	0.02	Nil result
			Mean Diff (%)	-4.36 (54.5%)	-4.24 (53.0%)	Nil result
	4.1	Observer A	Mean (SD) kPa	10.84 (0.39)	10.70 (0.21)	Nil result
			CV	0.04	0.02	Nil result
			Mean Diff (%)	-2.84 (35.5%)	-2.70 (33.8%)	Nil result
		Observer B	Mean (SD) kPa	9.79 (0.16)	9.69 (0.15)	Nil result
			CV	0.01	0.02	Nil result
			Mean Diff (%)	-1.79 (22.4%)	-1.69 (21.1%)	Nil result
	6.5	Observer A	Mean (SD) kPa	7.25 (0.39)	8.02 (0.15)	6.30 (0.41)
			CV	0.05	0.02	0.06
			Mean Diff (%)	0.75 (-9.4%)	-0.02 (0.3%)	1.70 (-21.3%)
		Observer B	Mean (SD) kPa	6.86 (0.20)	7.32 (0.08)	5.71 (0.13)
			CV	0.03	0.01	0.02
			Mean Diff (%)	1.14 (-14.3%)	0.68 (-8.5%)	2.29 (-28.6%)
	10.4	Observer A	Mean (SD) kPa	5.77 (0.36)	6.78 (0.10)	5.23 (0.10)
			CV	0.06	0.01	0.02
			Mean Diff (%)	2.23 (-27.9%)	1.22 (-15.3%)	2.77 (-34.6%)
		Observer B	Mean (SD) kPa	5.89 (0.14)	6.77 (0.10)	4.72 (0.10)
			CV	0.02	0.01	0.02
			Mean Diff (%)	2.11 (-26.4%)	1.23 (-15.4%)	3.28 (-41.0%)
	16.7	Observer A	Mean (SD) kPa	5.16 (0.15)	6.41 (0.06)	4.75 (0.16)
			CV	0.03	0.01	0.03
			Mean Diff (%)	2.84 (-35.5%)	1.59 (-19.9%)	3.25 (-40.6%)
		Observer B	Mean (SD) kPa	6.06 (0.82)	6.00 (0.04)	4.52 (0.07)
			CV	0.14	0.01	0.02
			Mean Diff (%)	1.94 (-25.3%)	2.00 (-25.0%)	3.48 (-43.5%)
Type 2 (14 kPa)	2.5	Observer A	Mean (SD) kPa	12.36 (0.44)	12.93 (0.46)	Nil result
			CV	0.04	0.04	Nil result
			Mean Diff (%)	1.64 (-11.7%)	1.07 (-7.6%)	Nil result
		Observer B	Mean (SD) kPa	12.09 (0.44)	12.30 (0.23)	Nil result
			CV	0.04	0.02	Nil result
			Mean Diff (%)	1.91 (-13.6%)	1.7 (-12.1%)	Nil result
	4.1	Observer A	Mean (SD) kPa	12.31 (0.25)	12.71 (0.11)	Nil result
			CV	0.02	0.01	Nil result
			Mean Diff (%)	1.69 (-12.1%)	1.29 (-9.2%)	Nil result
		Observer B	Mean (SD) kPa	11.70 (0.45)	10.99 (0.13)	Nil result
			CV	0.04	0.01	Nil result
			Mean Diff (%)	2.30 (-16.4%)	3.01 (-21.5%)	Nil result
	6.5	Observer A	Mean (SD) kPa	11.29 (0.17)	11.26 (0.12)	10.37 (0.22)
			CV	0.02	0.01	0.02
			Mean Diff (%)	2.71 (-19.4%)	2.74 (-19.6%)	3.63 (-25.9%)
		Observer B	Mean (SD) kPa	10.25 (0.40)	10.42 (0.04)	9.84 (0.20)
			CV	0.04	0.00	0.02
			Mean Diff (%)	3.75 (-26.8%)	3.58 (-25.6%)	4.16 (-29.7%)
	10.4	Observer A	Mean (SD) kPa	10.34 (0.13)	11.01 (0.60)	10.20 (0.20)
			CV	0.01	0.01	0.02
			Mean Diff (%)	3.66 (-26.1%)	2.99 (-21.4%)	3.8 (-27.1%)
		Observer B	Mean (SD) kPa	9.95 (0.17)	10.11 (0.04)	9.37 (0.19)
			CV	0.02	0.00	0.02
			Mean Diff (%)	4.05 (-28.9%)	3.89 (-27.8%)	4.63 (-33.1%)
	16.7	Observer A	Mean (SD) kPa	9.71 (0.07)	9.78 (0.04)	9.99 (0.14)
			CV	0.01	0.00	0.01
			Mean Diff (%)	4.29 (-30.6%)	4.22 (-30.1%)	4.01 (-28.6%)
		Observer B	Mean (SD) kPa	9.09 (0.06)	9.76 (0.06)	8.94 (0.22)
			CV	0.01	0.01	0.03
			Mean Diff (%)	4.91 (-35.1%)	4.24 (-30.3%)	5.06 (-36.1%)
Type 3 (45 kPa)	2.5	Observer A	Mean (SD) kPa	19.24 (0.37)	13.91 (0.11)	Nil result
			CV	0.02	0.01	Nil result
			Mean Diff (%)	25.76 (-57.2%)	31.09 (-69.1%)	Nil result
		Observer B	Mean (SD) kPa	13.80 (0.24)	10.98 (0.13)	Nil result
			CV	0.02	0.01	Nil result
			Mean Diff (%)	31.20 (-69.3%)	34.02 (-75.6%)	Nil result
	4.1	Observer A	Mean (SD) kPa	15.28 (0.28)	17.58 (0.10)	Nil result
			CV	0.02	0.01	Nil result
			Mean Diff (%)	29.72 (-66.0%)	27.42 (-60.9%)	Nil result
		Observer B	Mean (SD) kPa	14.44 (0.27)	20.19 (0.50)	Nil result
			CV	0.02	0.03	Nil result
			Mean Diff (%)	30.56 (-67.9%)	24.81 (-55.1%)	Nil result
	6.5	Observer A	Mean (SD) kPa	21.69 (0.17)	24.86 (0.21)	17.92 (0.82)
			CV	0.01	0.01	0.01
			Mean Diff (%)	23.31 (-51.8%)	20.14 (-44.8%)	27.08 (-60.2%)
		Observer B	Mean (SD) kPa	22.34 (0.53)	23.83 (0.48)	17.22 (0.79)
			CV	0.02	0.02	0.05
			Mean Diff (%)	22.66 (-50.4%)	21.17 (-47.0%)	27.78 (-61.7%)
	10.4	Observer A	Mean (SD) kPa	28.30 (0.69)	29.63 (0.28)	9.01 (1.07)
			CV	0.02	0.01	0.12
			Mean Diff (%)	16.7 (-37.1%)	15.37 (-34.2%)	35.99 (-80.0%)
		Observer B	Mean (SD) kPa	27.61 (0.36)	29.98 (0.55)	9.06 (2.41)
			CV	0.01	0.02	0.27
			Mean Diff (%)	17.39 (-38.6%)	15.02 (-33.4%)	35.94 (79.9%)
	16.7	Observer A	Mean (SD) kPa	30.91 (0.41)	32.93 (0.17)	25.81 (1.24)
			CV	0.01	0.01	0.05
			Mean Diff (%)	14.09 (-31.3%)	12.07 (-26.8%)	19.19 (-42.6%)
		Observer B	Mean (SD) kPa	31.19 (0.32)	31.98 (0.23)	25.97 (0.58)
			CV	0.01	0.01	0.02
			Mean Diff (%)	13.81 (-30.7%)	13.02 (-28.9%)	19.03 (-42.3%)
Type 4 (80 kPa)	2.5	Observer A	Mean (SD) kPa	20.39 (0.61)	20.08 (0.89)	Nil result
			CV	0.03	0.04	Nil result
			Mean Diff (%)	59.61 (-74.5%)	59.92 (-74.9%)	Nil result
		Observer B	Mean (SD) kPa	19.86 (0.76)	20.35 (0.21)	Nil result
			CV	0.04	0.01	Nil result
			Mean Diff (%)	60.14 (-75.2)	59.65 (-74.6%)	Nil result
	4.1	Observer A	Mean (SD) kPa	25.66 (1.35)	29.29 (0.76)	Nil result
			CV	0.05	0.03	Nil result
			Mean Diff (%)	54.34 (-67.9%)	50.71 (-63.4%)	Nil result
		Observer B	Mean (SD) kPa	24.63 (0.57)	25.82 (0.65)	Nil result
			CV	0.02	0.03	Nil result
			Mean Diff (%)	55.37 (-69.2%)	54.18 (-67.7%)	Nil result
	6.5	Observer A	Mean (SD) kPa	36.14 (0.83)	43.39 (0.818)	11.09 (0.99)
			CV	0.02	0.02	0.09
			Mean Diff (%)	43.86 (-54.8%)	36.61 (-45.8%)	68.91 (-86.1%)
		Observer B	Mean (SD) kPa	38.72 (0.78)	41.53 (0.85)	12.40 (0.77)
			CV	0.02	0.02	0.06
			Mean Diff (%)	41.28 (-51.6%)	38.47 (-48.1%)	67.60 (-84.5%)
	10.4	Observer A	Mean (SD) kPa	45.36 (1.98)	52.44 (0.41)	9.50 (1.43)
			CV	0.04	0.01	0.15
			Mean Diff (%)	34.64 (-43.3%)	27.56 (-30.7%)	70.5 (-88.1%)
		Observer B	Mean (SD) kPa	45.57 (1.18)	53.90 (0.72)	8.78 (1.52)
			CV	0.03	0.01	0.17
			Mean Diff (%)	34.43 (-43.0%)	26.1 (-32.6%)	71.22 (89.0%)
	16.7	Observer A	Mean (SD) kPa	52.39 (1.22)	64.88 (0.42)	23.68 (3.90)
			CV	0.02	0.01	0.16
			Mean Diff (%)	27.61 (-34.5%)	15.12 (-18.9%)	56.32 (-70.4%)
		Observer B	Mean (SD) kPa	50.24 (0.23)	60.71 (0.69)	28.96 (18.88)
			CV	0.01	0.01	0.65
			Mean Diff (%)	29.76 (-37.2%)	19.29 (-24.1%)	51.04 (-63.8%)
